# Live imaging of cortical granule exocytosis reveals that *in vitro* matured mouse oocytes are not fully competent to secrete their content

**DOI:** 10.1242/bio.031872

**Published:** 2018-12-15

**Authors:** Andrea I. Cappa, Matilde de Paola, Paula Wetten, Gerardo A. De Blas, Marcela A. Michaut

**Affiliations:** 1 Laboratorio de Biología Reproductiva y Molecular, Instituto de Histología y Embriología, Universidad Nacional de Cuyo-CONICET, Av. Libertador 80, 5500, Mendoza, Argentina; 2 Universidad Nacional de Cuyo, Facultad de Ciencias Médicas, Área de Farmacología, Av. Libertador 80, 5500, Mendoza, Argentina; 3 Universidad Nacional de Cuyo, Facultad de Ciencias Exactas y Naturales, Departamento de Biología, Padre Jorge Contreras 1300, 5500, Mendoza, Argentina

**Keywords:** Cortical granule exocytosis, Real time, *In vitro* maturation, Mouse oocyte, Live imaging, Cortical reaction

## Abstract

Oocyte *in vitro* maturation does not entirely support all the nuclear and cytoplasmic changes that occur physiologically, and it is poorly understood whether *in vitro* maturation affects the competence of cortical granules to secrete their content during cortical reaction. Here, we characterize cortical granule exocytosis (CGE) in live mouse oocytes activated by strontium chloride using the fluorescent lectin FITC-LCA. We compared the kinetic of CGE between ovulated (*in vivo* matured, IVO) and *in vitro* matured (IVM) mouse oocytes. Results show that: (1) IVM oocytes have a severely reduced response to strontium chloride; (2) the low response was confirmed by quantification of remnant cortical granules in permeabilized cells and by a novel method to quantify the exudate in non-permeabilized cells; (3) the kinetic of CGE in IVO oocytes was rapid and synchronous; (4) the kinetic of CGE in IVM oocytes was delayed and asynchronous; (5) cortical granules in IVM oocytes show an irregular limit in regards to the cortical granule free domain. We propose the analysis of CGE in live oocytes as a biological test to evaluate the competence of IVM mouse oocytes.

This article has an associated First Person interview with the first author of the paper.

## Abstract (precis)

**Summary:** Live imaging of cortical granule exocytosis reveals that *in vitro* matured mouse oocytes have a severely reduced response to strontium chloride.

## INTRODUCTION

In mammalian oocytes, cortical reaction, also named cortical granule exocytosis (CGE), is a fundamental process in which the cortical granules fuse with the plasma membrane after sperm fertilization preventing polyspermy and ensuring embryo development [reviewed by Liu (2011); Sun (2003)]. The production of cortical granules in mammalian oocytes is a continuous process, and newly synthesized granules are translocated to the cortex until the time of ovulation (Ducibella et al., 1994). The migration of cortical granules to the cortex is mediated by microfilaments (Cheeseman et al., 2016; Connors et al., 1998) and is an important step in cytoplasmic maturation (Ducibella et al., 1988a). The localization of cortical granules in the cortical region is used routinely as a criterion in assessing the maturity and organelle organization of developing oocytes (Damiani et al., 1996).

Oocyte meiotic maturation is a complex process that involves coordinated nuclear and cytoplasmic changes and is defined as the resumption and completion of the first meiotic division up until metaphase II. The completion of nuclear and cytoplasmic processes defines the competence of an oocyte. Only a competent oocyte can be fertilized and support early embryo development (Li and Albertini, 2013). The underlying cellular and molecular mechanisms of mammalian oocyte maturation are still poorly understood and are under continuous investigation (Reader et al., 2017).

*In vitro* maturation (IVM) is a culture method that allows germinal vesicle (GV) oocytes to undergo IVM until reaching metaphase II stage (MII oocytes). IVM is used in both animal and human assisted reproduction, but the reproductive efficiency is very low. Cortical granules become fully competent for exocytosis after completion of the first meiotic division in MII oocytes (Ducibella et al., 1988b; Ducibella and Buetow, 1994). How IVM affects the competence of cortical granules to secrete their content is under continuous investigation. In this report, we investigated the reaction capacity to strontium chloride (SrCl_2_) of *in vivo* (IVO) and *in vitro* matured (IVM) oocytes, using a fluorescent method to analyze CGE in real time.

## RESULTS

### The dynamics of cortical reaction can be evaluated in real time by LCA-FITC

The distribution of cortical granules in rodents MII oocytes has been demonstrated using fluorescence microscopy with the fluorescently labeled lectin *Lens culinaris* agglutinin (LCA) (Cherr et al., 1988; Ducibella et al., 1988a). Cherr and collaborators demonstrated that LCA allows the localization of cortical granule content before and after exocytosis in hamster MII oocytes (Cherr et al., 1988). LCA-FITC has an affinity with alpha-mannose residues present in the content of cortical granules. When this content is secreted during CGE, the secretion can be detected by fluorescence microscopy. Hence, we use LCA-FITC to analyze CGE in real time. First, we attempted to activate CGE with mouse sperm by *in vitro* fertilization. Unfortunately, this method was impracticable because mouse sperm agglutinated in presence of LCA-FITC (see Movie 1). Then, we decided to activate CGE parthenogenetically with SrCl_2_. This parthenogenetic activator has several advantages compared to other chemical and physical activators; its use is very simple, it is not toxic for the cell, it mimics the natural pattern of calcium waves after sperm penetration, and it synchronizes cortical reaction in most of the oocytes within a relatively short time frame. This is a significant advantage over using fertilized eggs, which are typically activated at various times throughout an experiment. In addition, artificial activation alleviates concerns of having to differentiate sperm-derived and egg-derived constituents.

**Fig. 1.**
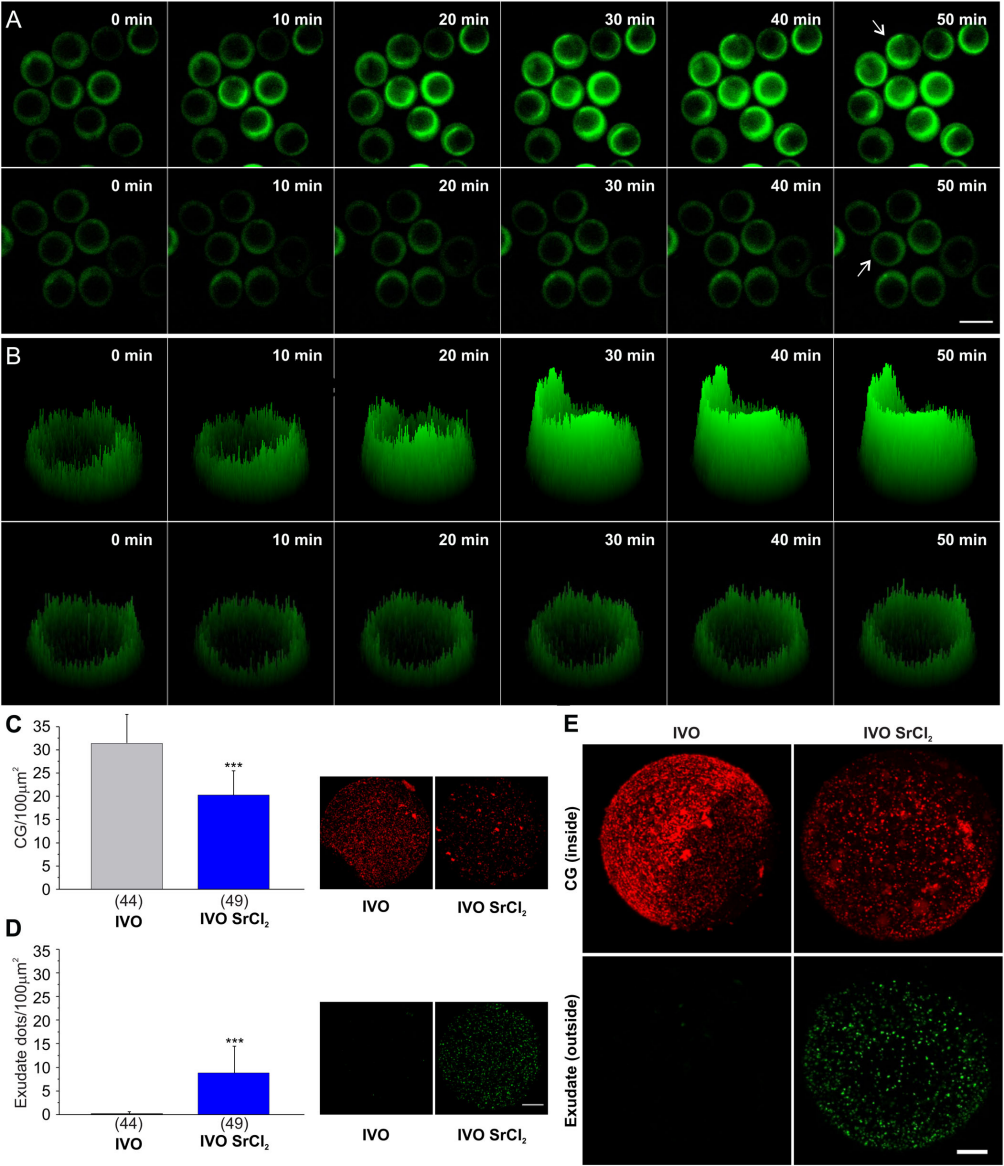
**Cortical reaction in real time.** (A) Epifluorescence microscopy images of *in vivo* ovulated (IVO) oocytes population incubated with (up) or without (down) SrCl_2_ in presence of LCA-FITC. Scale bar: 100 µm. (B) 3D Surface plot of the oocyte indicated by arrow in A: oocyte incubated with (up) or without (down) SrCl_2_. (C) Cortical granule density per 100 μm^2^ (CG/100 μm^2^) in IVO oocytes activated (blue bar) or not activated (gray bar) with SrCl_2_. On the right side of the bar chart, representative confocal images of cortical granules are shown. (D) Exudate dots density per 100 μm^2^ and representative confocal images of exudate from the same oocytes evaluated in C. Data in C and D are shown as mean±s.d. from at least three independent experiments. Numbers in parentheses represent total number of oocytes. Asterisks indicate statistically significant differences between groups (*P*<0.05, Student's *t*-test). Scale bar: 20 µm. (E) 3D reconstruction from multiple confocal images of two representative IVO oocytes showing cortical granules (up) and exudate (down) in the presence (right) or absence (left) of SrCl_2_. Scale bar: 10 µm. Red color (LCA-rhodamine) shows cortical granules that remain in the cell (CG inside). Green color (LCA-FITC) shows exudate dots produced by the release of cortical granule content in the presence of LCA-FITC after cell fixation (exudate outside). Images of IVO condition correspond to the same oocyte; the same for IVO SrCl_2_.

**Fig. 2.**
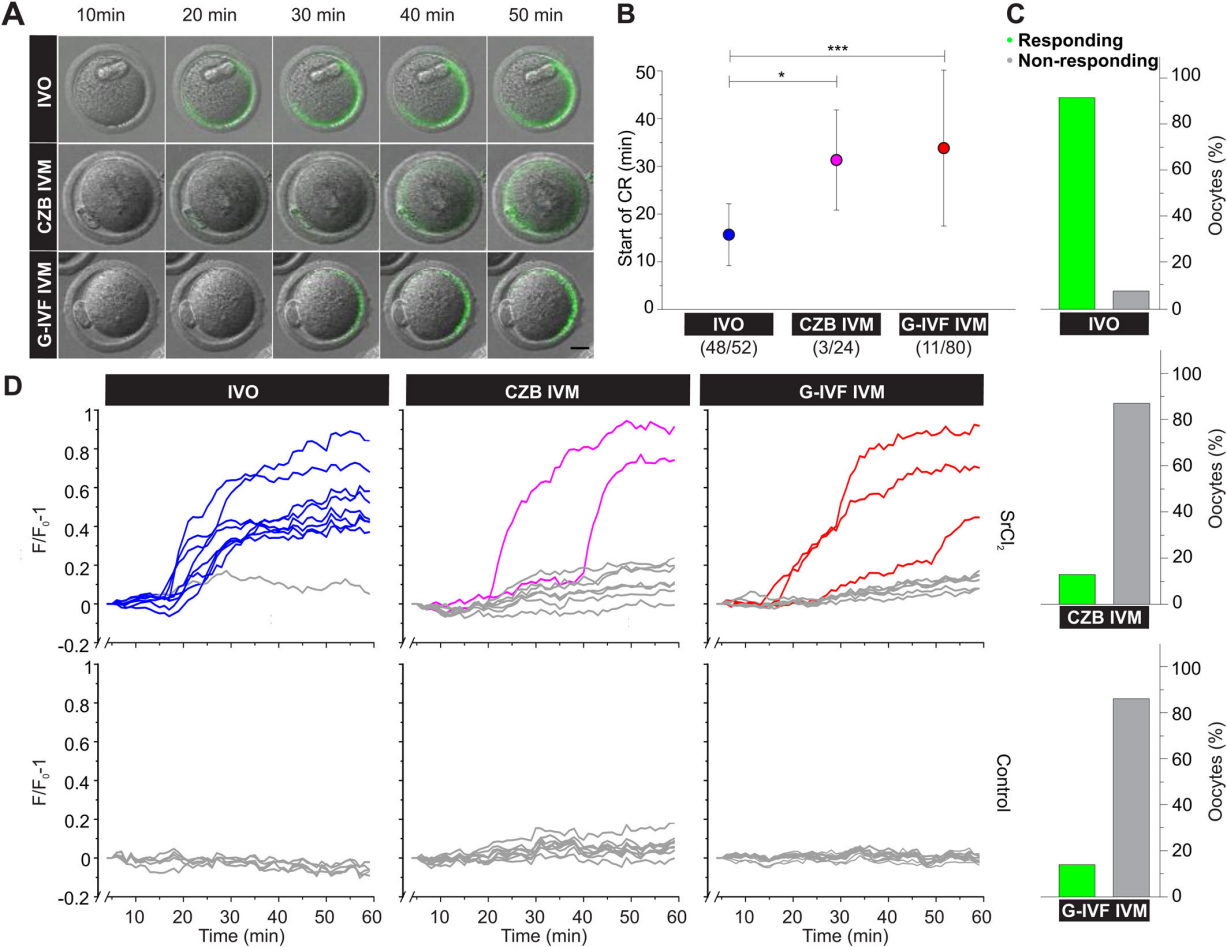
**Live imaging of cortical reaction in ovulated oocytes (IVO) and *in vitro* matured (IVM) oocytes.** (A) Merged images from FV1000 Olympus confocal microscope from DIC and FITC channels showing a representative SrCl_2_-activated oocyte in the presence of LCA-FITC for IVO, CZB IVM and G-IVF IVM oocytes. Scale bar: 20 µm. (B) Start time of cortical reaction (CR) represented as mean±s.d. from at least three independent experiments. Numbers in parentheses represent number of responding oocytes from total. Asterisks indicate statistically significant differences between groups (*P< 0.05; ***P< 0.001) (*P*<0.05, Tukey's test). (C) Percentage of responding (green bar) and non-responding oocytes (grey bar) under SrCl_2_ stimulation in the same experiments evaluated in B. Top to bottom: IVO (*n*=52), CZB IVM (*n*=24) and G-IVF IVM (*n*=80) oocytes. Percentages of IVM responding oocytes (green bars) were significantly different compared to IVO responding oocytes (*P*<0.0001, test of difference between two proportions). (D) Kinetic of cortical reaction. Fluorescence increase represented as (F/F_0_-1) versus time from IVO, CZB IVM and G-IVF IVM oocytes incubated with (up) or without (down) SrCl_2_ for 1 h in the presence of LCA-FITC. Gray lines represent non-responding oocytes and color lines represent responding oocytes.

We incubated IVO oocytes in the presence of LCA-FITC to detect the secretion of cortical granules into the perivitelline space by the increase of fluorescence. The activator was present in the incubation media during the entire experiment. Only MII oocytes showing the first polar body were used for the assay. As shown in Fig. 1A (upper panel), we were able to detect a visible increase of LCA-FITC intensity after 10–20 min of the addition of strontium chloride (see also Movie 2). The fluorescence's intensity was irregular. It drew a fluorescent semicircle with high intensity and a small portion of the circle with low intensity, resembling the localization of cortical granules (vegetal pole) and the cortical granules free domain (animal pole), respectively (Fig. 1B, compare upper and lower panels at 50 min, also see Fig. 2A, IVO panel). In contrast, control (not activated) MII oocytes did not show any fluorescence increase during the recorded time (lower panel in Fig. 1A,B; and Movie 3).

In accordance with the literature, the observed time correlates with the timing of CGE in MII mouse oocytes (Liu, 2011). To demonstrate that the fluorescence increase corresponded effectively to the release of cortical granules, the cells were pooled and fixed for cortical granule quantification as we previously described (Bello et al., 2016; de Paola et al., 2015). After fixation, cells were permeabilized and the remnant of cortical granules was stained with LCA-rhodamine. Using the program ImageJ, the density of cortical granules (in the same oocytes analyzed in the live condition) was quantified as a measurement of cortical reaction. As predicted, the density of remnant cortical granules (represented as CG/100 µm^2^) was significantly lower in oocytes treated with SrCl_2_ than in control oocytes (Fig. 1C). This result indicates that cortical granules fused with the plasma membrane and secreted their content into the perivitelline space.

Cherr and collaborators have demonstrated by transmission electronic microscopy that gold-LCA binds to the microvilli of the activated zona-free hamster eggs (Cherr et al., 1988). In fact, as a consequence of the activation of live MII oocytes in presence of LCA-FITC, the exterior of the cells was also stained by this fluorescent lectin after cell fixation. We observed by confocal microscopy that LCA-FITC (used to stain the secretion of cortical granules in live oocytes), also drew a punctuate pattern in the outside of the fixed oocytes. This punctuate pattern was suitable to be quantified in a similar manner to cortical granules quantification. We named this pattern ‘exudate dots’. We quantified the dots’ density and found that this punctuate pattern can also be used to measure the magnitude of cortical reaction (Fig. 1D–E). Fig. 1E shows representative images for control (left column, IVO, not activated) and activated (right column, IVO SrC_2_) oocytes. In the control condition, cortical granules remain inside of the cell and no cortical reaction is observed. Thus, the upper panel of Fig. 1E (left) shows cortical granules stained with LCA-rodhamine in a control oocyte (CG ‘inside’). Without activation and incubated in presence of LCA-FITC, no cortical reaction is observed. This is evidenced by the absence of LCA-FITC dots in the exudate of the same oocyte (lower panel, left, exudate ‘outside’). On the contrary, in the activated condition the content of cortical granules was secreted and stained by the fluorescent LCA during live imaging. The upper panel of Fig. 1E (right) shows the remnant cortical granule after strontium activation (CG ‘inside’) and the exudates’ dots stained with LCA-FITC (lower panel, exudate ‘outside’) for the same oocyte. Finally, to validate LCA-FITC staining in a physiological context, we performed *in vitro* fertilization with a modified protocol to avoid spermatozoa agglutination. Oocytes were inseminated with capacitated spermatozoa in absence of LCA-FITC. After 2 h, embryos were transferred to an LCA-FITC-supplemented medium and incubated for additional 5 h. As shown in Fig. S1, *in vitro* fertilized oocytes showed a similar pattern of exudate in live and fixed cells as the pattern observed in strontium activated oocytes. Altogether, these experiments demonstrate that the increase of fluorescence intensity corresponds to the secretion of cortical granule content. Hence, the dynamics of CGE can be evaluated in live cells by analyzing the increase of LCA-FITC fluorescence intensity accumulated in the perivitelline space through time. Additionally, this approach also allows quantifying exudate dots in non permeabilized oocytes after live imaging.

### Live imaging of cortical reaction in ovulated and *in vitro* matured mouse oocytes

Injection of germinal vesicle-intact oocytes (GV oocytes) with mRNA is routinely used to study the role of different proteins in oocyte maturation. However, how closely *in vitro* maturation resembles the *in vivo* process and what the impact of *in vitro* maturation is on cortical granules releasing capacity remains unexplored. To analyze whether *in vitro* maturation has any impact on CGE, we *in vitro* matured mouse oocytes in two different media: CZB, a regular medium used for *in vitro* mouse oocyte maturation, and G-IVF, a medium used for human oocytes. Only cumulus-oocyte complexes were selected for IVM assays. First, we compared the morphology of IVM oocytes in either CZB or G-IVF medium with IVO oocytes. Results showed that both media supported *in vitro* oocyte maturation in a similar percentage. The percentage of IVM oocytes, quantified morphologically by the extrusion of the first polar body, was 64.4±14.49% in CZB (*n*=97) and 74.5±7.08% in G-IVF (*n*=325). Next, we assayed CGE in live cells using the method described in the previous section. We incubated IVO and IVM oocytes in the presence of LCA-FITC and analyzed the increase of fluorescence intensity in the perivitelline space after triggering CGE with SrCl_2_ for 60 min. As shown in Fig. 2A, IVO oocytes initiated CGE before IVM oocytes. CGE in IVO oocytes started around 16 min after SrCl_2_ addition (16±6 min, see IVO condition in Fig. 2B and Movie 4). Surprisingly, only a low percentage of IVM oocytes were able to respond to the parthenogenetic activator. Fig. 2C shows that only 13% of oocytes matured in CZB and 14% of oocytes matured in G-IVF medium were able to respond to SrCl_2_ (see Movies 5,6), while 92% of IVO oocytes responded to the stimulus. These percentages were significantly different compared to responding IVO oocytes (*P*<0.0001, test of difference between two proportions). In addition, IVM delayed the start time of CGE (Fig. 2A,B). IVM oocytes responded around 30–35 min after the addition of strontium chloride (31±11 min in CZB and 34±16 min in G-IVF). We wondered whether those oocytes that did not respond within 60 min would be activated in a prolonged assay. When the live imaging was extended until 120–180 min, we observed that IVM oocytes were not activated by SrCl_2_ (absence of cortical reaction and parthenogenetic development; data not shown). These results indicate that most IVM oocytes are not able to respond to SrCl_2_. Fig. 2D shows the kinetic of cortical reaction for IVO and IVM oocytes. IVO oocytes showed a rapid and synchronous response to SrCl_2_. However, IVM oocytes presented a later and asynchronous response to the activator of CGE (Fig. 2D). This asynchronous response is evidenced, mathematically, by a wider standard deviation in the start time of CGE in IVM oocytes (in both media) compared to IVO oocytes (Fig. 2B).

**Fig. 3.**
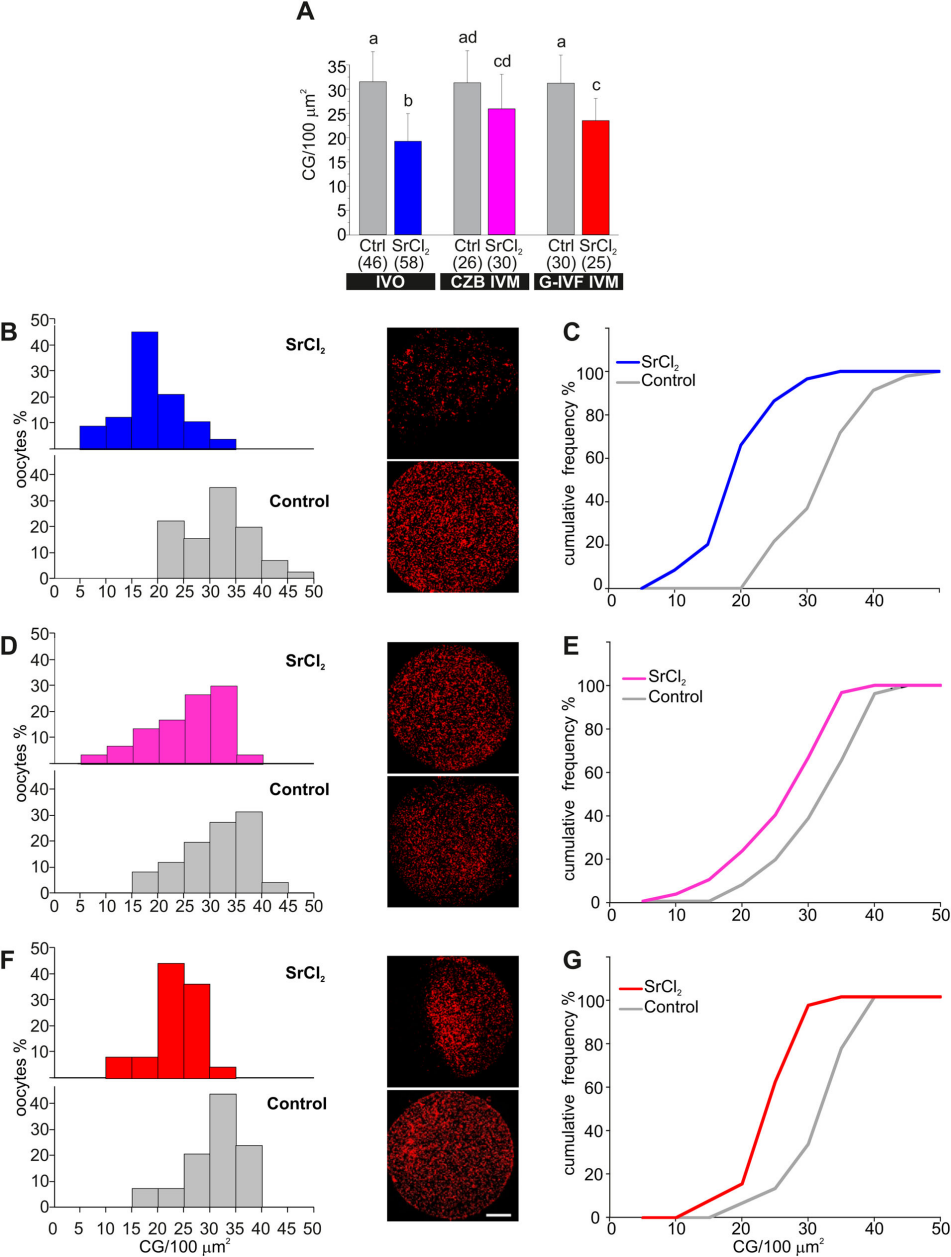
**Quantification of cortical granule (CG) exocytosis in fixed oocytes.** (A) Bar graph of cortical granules density (CG/100 μm^2^) of treated oocytes with (SrCl_2_) or without SrCl_2_ (Ctrl) for each condition: *in vivo* ovulated (IVO), CZB *in vitro* matured (CZB IVM) and G-IVF *in vitro* matured (G-IVF IVM) oocytes. Data are shown as mean±s.d. from at least three independent experiments; numbers in parentheses represent the total number of evaluated oocytes. Different letters indicate statistically differences between groups (*P*≤0.05, Steel–Dwass test). (B,D,F) Relative frequency histograms of cortical granule density for the same oocytes analyzed in A. Representative confocal images of oocytes subjected to each treatment are shown on the right. Cortical granules were labeled with LCA-rhodamine. Scale bar: 20 μm. (C,E,G) Cumulative relative frequency of cortical granule density for the same oocytes analyzed in A.

**Fig. 4.**
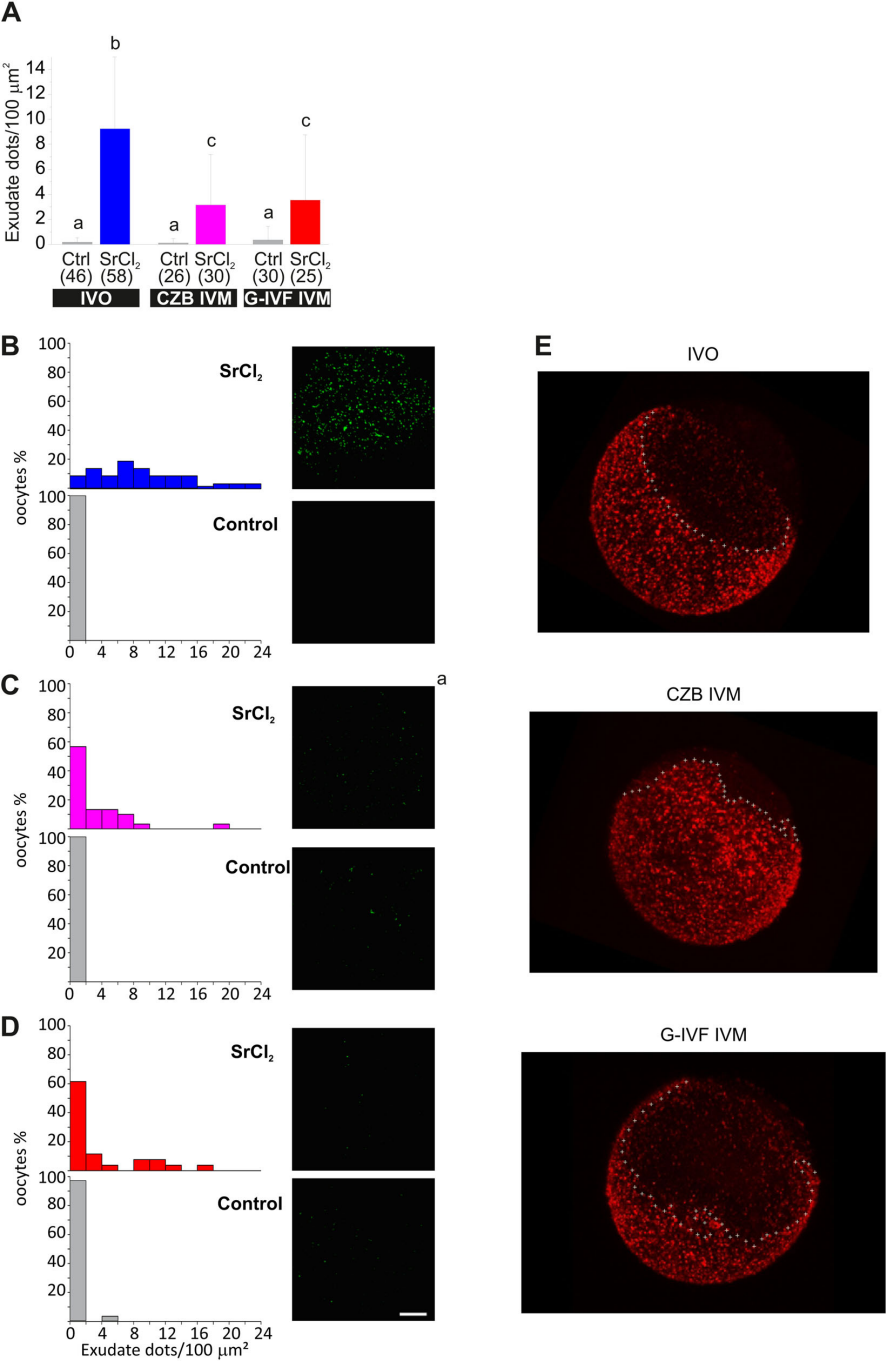
**Quantification of exudate dots in fixed oocytes and cortical granule localization.** (A) Bar graph of exudate dots density per 100 μm^2^ of oocytes analyzed in Fig. 3. Data are shown as mean±s.d. from at least three independent experiments; numbers in parentheses represent the total number of evaluated oocytes. Different letters indicate statistically significant differences between groups (*P*<0.05, Steel–Dwass test). (B,C,D) Relative frequency histograms of exudate dot density for the same oocytes analyzed in A. Representative confocal images of the same oocytes from Fig. 3 are shown on the right, now showing exudate labeled with LCA-FITC. Scale bar: 20 μm. (E) Distribution of cortical granules. 3D reconstruction from multiple confocal images of representative oocytes showing cortical granules distribution labeled with LCA-rhodamine in ovulated and *in vitro* matured oocytes. IVO, ovulated oocyte; CZB IVM, CZB *in vitro* matured oocyte; G-IVF IVM, G-IVF *in vitro* matured oocyte.

Then, after each experiment, cells were pooled at the end of the imaging session and fixed for cortical granule staining with LCA-rhodamine. The magnitude of CGE was analyzed in the whole cohort of imaged oocytes by quantification of density of remnant cortical granules. Fig. 3A shows that IVO oocytes responded in a higher magnitude that IVM cells (compare blue, pink and red bars). In effect, IVO oocytes secreted around 40% of cortical granules when stimulated by SrCl_2_ (blue bar), while IVM oocytes only released 17% (CZB IVM, pink bar) and 25% (G-IVF IVM, red bar) of granules. To better understand oocyte distribution, cells were grouped according to similar cortical granule density and plotted as histograms (Fig. 3B,D,F). In control conditions, most of the cells had more than 20 CG/100 µm^2^ (see gray bars and gray line in Fig. 3B and C, respectively). After stimulation with SrCl_2_, 70% of IVO oocytes had less than 20 CG/100 µm^2^ (average for IVO oocytes) (see blue bars in Fig. 3B and blue lines in Fig. 3C). This means that most of the cells responded to the activator and secreted the content of cortical granules. In contrast, when IVM oocytes were stimulated, only 20–30% of cells showed less than 20 CG/100 µm^2^ (see Fig. 3D–E and F–G), indicating that most of IVM oocytes were not able to secrete cortical granule content. Similar results were obtained by quantifying exudate dots in the same oocytes (Fig. 4). Fig. 4B shows that the exudate in non-stimulated IVO oocytes (control condition, gray bar) has between 0–2 exudate dots/100 µm^2^. On the contrary, when IVO oocytes were activated with SrCl_2_, around 80% of the cells showed more than 4 exudate dots/100 µm^2^. We then considered this number as the minimum cortical granule density for an activated oocyte. When IVM oocytes were analyzed, both IVM conditions – CZB and G-IVF medium – showed that 70% of the cells had less than 4 exudate dots/100 µm^2^, indicating that most of the IVM oocytes were not activated by SrCl_2_ (see Fig. 4C,D).

It is known that localization of cortical granules is mainly cytoplasmic in GV oocytes, and during IVO maturation they migrate to the cortical region of MII stage. This migration and final localization determines that MII oocytes have two easily distinguishable poles: the cortical granules region (also named vegetal pole) and the cortical granules free domain (also named animal pole) (de Paola et al., 2015; Ducibella et al., 1988a). We wondered whether IVM may perturb cortical granule migration and compared cortical granule localization between IVO and IVM oocytes using 3D reconstruction from multiple images. Results showed that in both conditions cortical granules localized at the cortical region; however, the limit between the two poles was different. IVO oocytes had a sharp and defined boundary (Fig. 4E, IVO panel; sharp limit in 16 from 23 oocytes), while IVM oocytes showed an irregular limit with the cortical granule free domain (Fig. 4E, CZB panel, sharp limit in 0 from 7 oocytes; G-IVF panel, sharp limit in 4 from 19 oocytes).

These results suggested that IVM may also perturb the molecular mechanism involved in the correct migration and/or localization of cortical granules.

## DISCUSSION

In this study, we evaluated CGE in real time using the fluorescent lectin FITC-LCA. A similar approach has been reported by Satouh and collaborators (Satouh et al., 2017). We investigated the capacity of reacting to SrCl_2_ of IVO and IVM oocytes. We found that, even when IVM oocytes had a normal morphology, they responded in a very low percentage compared to IVO oocytes. The low response was confirmed by quantification of remnant cortical granules in permeabilized cells and by a novel method to quantify the exudate dots in non permeabilized cells. The kinetic of CGE in IVO oocytes was rapid and synchronous. In contrast, it was delayed and asynchronous in IVM oocytes. Cortical granule distribution in IVM oocytes shows an irregular limit with the cortical granule free domain.

Why *in vitro* maturation alters the competence of cortical granules to secrete their content is still an unanswered question. The membrane fusion during this particular secretory process in mouse oocyte is thought to be mediated by SNAREs (soluble N-ethylmaleimide-sensitive factor attachment protein receptor) and other regulatory proteins. In effect, it has been shown that the SNAREs SNAP-25 (Ikebuchi et al., 1998) and Sintaxina 4 (Iwahashi et al., 2003) are involved in cortical granule exocytosis in IVO mouse oocytes. We have demonstrated that the alpha-SNAP/NSF complex regulates membrane fusion during cortical reaction and we have proposed a working model for cortical reaction in IVO mouse oocytes (de Paola et al., 2015). In addition, we also have characterized the participation of Rab3A in the cortical reaction of IVO mouse oocytes. Whether *in vitro* maturation disturbs the function of proteins involved in cortical granule exocytosis has not been yet investigated. Tsai and collaborators have shown that maturation-dependent migration of SNARE proteins, clathrin, and complexin to the porcine oocyte's surface blocks membrane traffic until fertilization (Tsai et al., 2011). Nevertheless, this general conclusion was reached using IVM oocytes. To the best of our knowledge, this is the first work that compares CGE in real time between IVO and IVM oocytes. Our findings invite to review the results and conclusions of the literature obtained with IVM oocytes since they should not be extrapolated to IVO oocytes.

The development of mouse oocyte cortical reaction competence during oocyte maturation between GV oocytes (incompetent) and MII oocytes (competent) is accompanied by morphological changes in cortical vesicles (Ducibella et al., 1988b). These changes have been associated with the correct maturation of calcium reservoirs such as endoplasmic reticulum (Kim et al., 2014; Latham, 2015), mitochondria (Dumollard et al., 2006), and probably cortical granules. Thus, calcium physiology and its reservoirs need to be explored in IVM oocytes in detail.

The localization of cortical granules in the cortical region is used as a criterion in assessing the maturity and organelle organization of developing mouse oocytes (Damiani et al., 1996). This work shows that cortical granules in IVM oocytes are localized in the cortical region; nevertheless, the limit between the animal and vegetal poles was different between IVO and IVM oocytes. While for IVO oocytes the boundary limit of cortical granules was well defined in 70% of cells, for IVM oocytes, this limit was sharp only for 0–21% of cells. It would be interesting to analyze the relationship between the irregular boundary of cortical granules and the successful activation of the oocyte. However, considering our findings show that IVM oocytes are not fully competent for cortical granules exocytosis, this analysis would only be possible having a transgenic mouse which *in vivo* expresses a fluorescent molecular marker on cortical granules.

Here, we are reporting evidence that demonstrates that the morphological observations alone are not a sufficient criterion to determine the competence of cortical granules and, in consequence, the oocyte's competence. Cortical reaction has been suggested as an oocyte quality indicator in pikeperch (Żarski et al., 2012). Satouh and collaborators have shown that the use of LCA-FITC is innocuous for pregnancy and delivery of mouse pups (Satouh et al., 2017). In this work, we have demonstrated that the pattern of LCA-FITC staining is similar in *in vitro* fertilized and parthenogenetic activated oocytes. Therefore, we propose the analysis of CGE in live mouse oocytes as a biological and innocuous test to determine the competence of a mouse oocyte (or early embryo). Knowing that IVM media does not entirely support all the nuclear and cytoplasmic changes that occur physiologically, this biological test would also allow evaluation of the capability of maturation media for supporting *in vitro* maturation.

## MATERIALS AND METHODS

The protocol was approved by the Institutional Animal Care and Use Committee of the School of Medicine of the National University of Cuyo (protocol approval 24/2014 and 52/2015).

### Reagents

All chemicals, unless stated otherwise, were purchased from Sigma-Aldrich Chemical Inc. (St. Louis, USA). All solutions were prepared with sterile and apyrogenic distilled water Roux-Ocefa (Bs. As., Argentina). G-IVF ™ PLUS and EmbryoMax^®^ CZB were from Vitrolife (Sweden) and Merk Millipore (USA), respectively. Pregnant mare's serum gonadotropin (PMSG) and human chorionic gonadotropin (hCG) were a generous gift from Syntex (Argentina). G-IVF, CZB and M16 medium drops (with/without milrinone) were always covered with mineral oil and gassed overnight at 37°C in a humidified atmosphere containing 5% (v/v) CO_2_ before use.

### Immature oocyte (GV) collection

All oocytes were obtained from CF-1 female mice between 6–12 weeks old. For follicular growth stimulation, female mice were injected with 10 IU PMSG (Syntex, Argentina). 43–45 h after PMSG injection, immature cumulus-oocyte complexes (COCs) were collected from the ovary in MEM/ HEPES (Minimum Essential Medium with 100 µg/ml sodium pyruvate, 10 µg/ml gentamicin, 25 mM HEPES pH 7.3) supplemented with milrinone (2.5 µM), which inhibits oocyte maturation. Then they were incubated, in the presence of milrinone, in drops of G-IVF or CZB medium until *in vitro* maturation.

### Mature oocyte (MII) collection

Females were injected with 10 IU hCG (Syntex, Argentina) 48 h after PMSG injection. MII oocytes were collected from ampulla 15–17 h later in MEM/HEPES medium. Cumulus cells were removed with a brief exposure to 0.04% hialuronidase and the oocytes were incubated in drops of M16 until partenogenetic stimulation.

### *In vitro* maturation

COCs were washed with three drops of either G-IVF or CZB medium without milrinone. Then they were incubated for 15–17 h in a final drop of 100 µl of the same medium.

### Cortical reaction in real time

Only oocytes with polar body were considered. In the case of *in vitro* matured oocytes, they were first emptied of cumulus cell by pipetting. No more than 10 MII oocytes were selected for each condition. They were quickly washed in two drops of Ca^2+^-free M2 medium supplemented with 25 µg/ml *Lens culinaris* Agglutinin (LCA)-FITC (Vector Laboratories, Burlingame, USA) with/without 30 mM SrCl_2_. Then they were placed in a round chamber containing a final drop of 50 µl of the same solution covered with mineral oil. Immediately, images were taken at 37°C every minute for 1 h using an inverted Eclipse TE300 Nikon microscope coupled to a Luca R EMCCD camera or a FV1000 Olympus confocal microscope.

### Cortical granule staining

After live imaging, the zona pellucida was removed by brief incubation in Tyrode's solution pH 2.2 and cells were fixed for 40 min in 3.7% paraformaldehyde in DPBS. They were washed in blocking solution (3 mg/ml BSA, 100 mM glycine and 0.01% Tween 20 in DPBS) for 15 min and then permeabilized with 0.1% Triton-X in DPBS for 15 min. Then, remnant cortical granules were stained by incubation with 25 µg/ml LCA-rhodamine (Vector Laboratories) in blocking solution for 30 min. Cells were washed again in blocking solution for 15 min and finally were mounted using Vectashield Mounting Medium (Vector Laboratories). For 3D reconstruction, cells were mounted, without being smashed, in a chamber with solid Vaseline around them.

### Image analysis

Cortical granule density per 100 μm^2^ for each cell was determined from confocal images as the mean of the counts from at least four non-overlapping equal areas of cortex containing cortical granules, according to our previous work (de Paola et al., 2015). Cortical granule density per 100 μm^2^ was calculated by computer-assisted image quantification using ImageJ. Exudate dots were quantified in the same way, using the same areas selected for cortical granule quantification. 3D reconstruction was performed using ‘3D Project’ plug-in from ImageJ (brightest point method) using single 2D confocal images taken every 2 µm along the z-axis. Surface plot was realized using the ‘Surface Plot’ command of ImageJ (polygon multiplier 100%). Kinetic of cortical reaction was measured from confocal images as fluorescent intensity (F) relative to baseline (F_0_) using the formula: F/F_0_-1, with the program ImageJ.

### Data analysis

Experiments were repeated at least three times. Data analysis was performed using KyPlot or Statgraphics software. The percentage of responding oocytes in each condition was compared using the test of difference between two proportions. Statistical significance of cortical granule density and exudate dot density was determined by Student's *t*-test, or Steel–Dwass test. The results are expressed as mean±s.d. *P*<0.05 was considered statistically significant.

## Supplementary Material

Supplementary information
