# Supplementary material for: Live imaging of cortical granule exocytosis reveals that in vitro matured mouse oocytes are not fully competent to secrete their content
Source: Biol Open. 2018 Dec 15;7(12):bio031872. doi: 10.1242/bio.031872 (PMC6310882; doi:10.1242/bio.031872)
Supplement: Supplementary information [file biolopen-7-031872-s1.pdf]

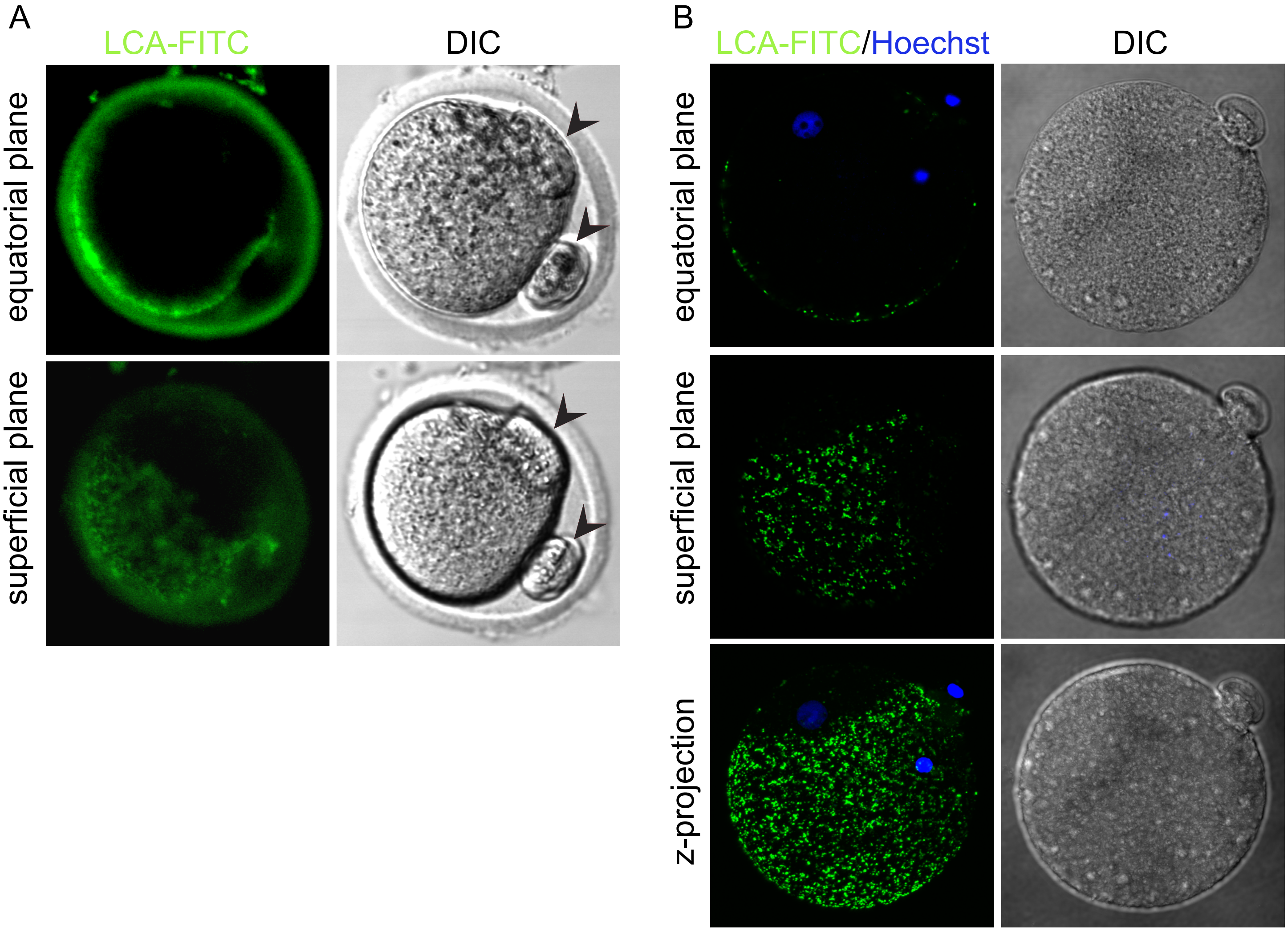

Figure S1. *In vitro* fertilized eggs stained with LCA-FITC. Eggs were inseminated with capacitated spermatozoa at a final concentration of 1-5 × 10^6 per ml for 2 hours. After washing, embryos were incubated in CZB medium supplemented with 5 µg/ml LCA–FITC for additional 5 hours. A. 1-cell embryo stained with LCA-FITC (live cell, confocal images); arrow heads indicate polar bodies. B. 1 cell embryo stained with LCA-FITC after fixation (fixed cell, confocal images and Z-projection of 3D reconstruction). Blue, DNA stained with Hoechst 3342. DIC, differential interference contrast.

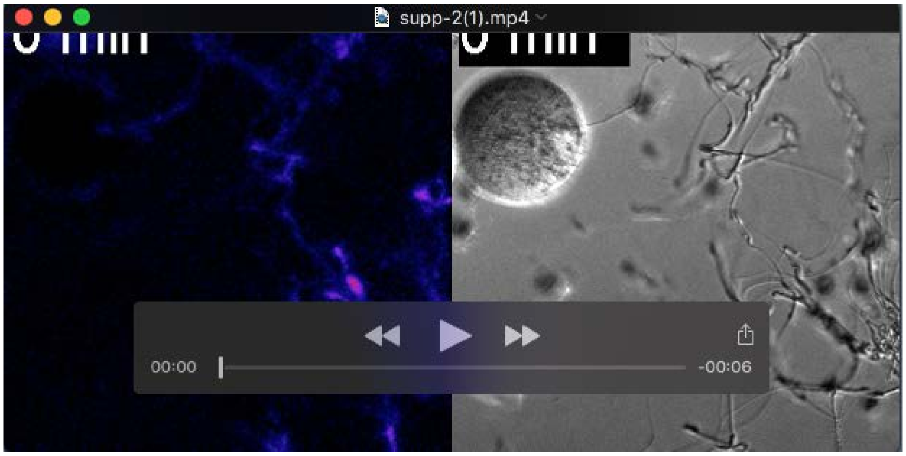

Movie 1. In vitro fertilization in presence of LCA-FITC: Confocal time lapse (left) and Differential interference contrast (DIC, right) microscopy of IVO oocytes incubated with mouse sperm in presence of fluorescenc lectin LCA-FITC.

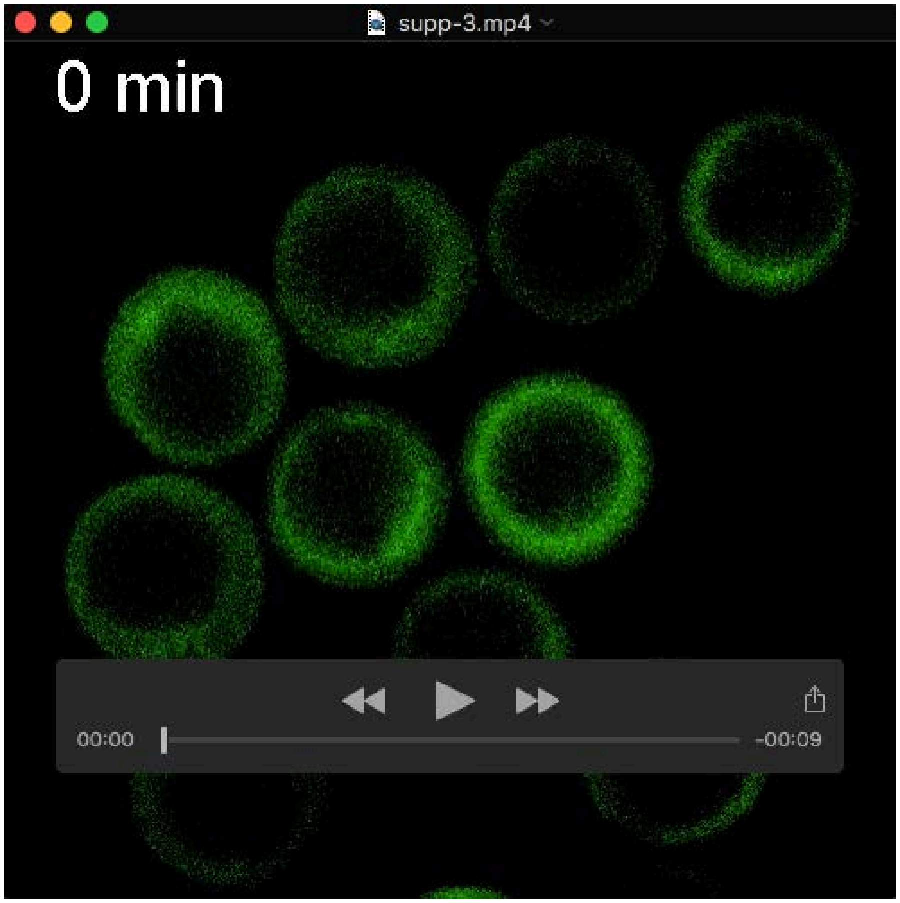

Movie 2. Live imaging of cortical reaction in ovulated oocytes: experimental condition. Epifluorescence time lapse of *in vivo* matured oocytes incubated with $SrCl_2$ in presence of fluorescent lectin LCA-FITC.

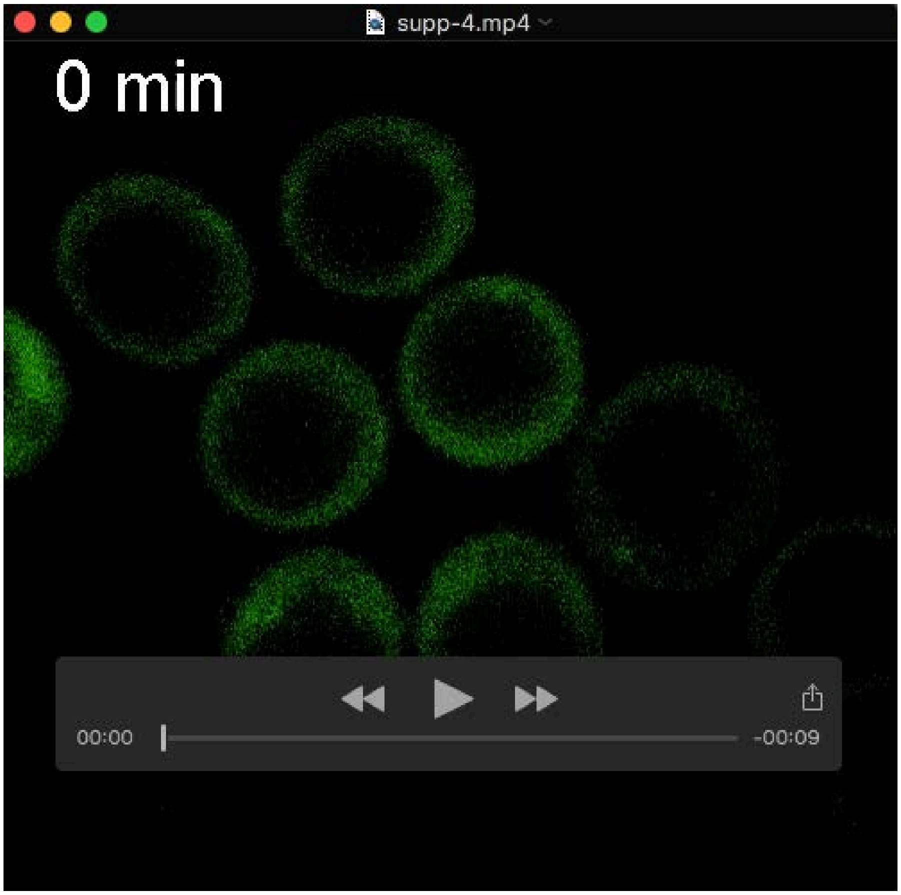

Movie 3. Live imaging of cortical reaction in ovulated oocytes: control condition. Epifluorescence time lapse of *in vivo* matured oocytes incubated without $SrCl_2$ in presence of LCA-FITC.

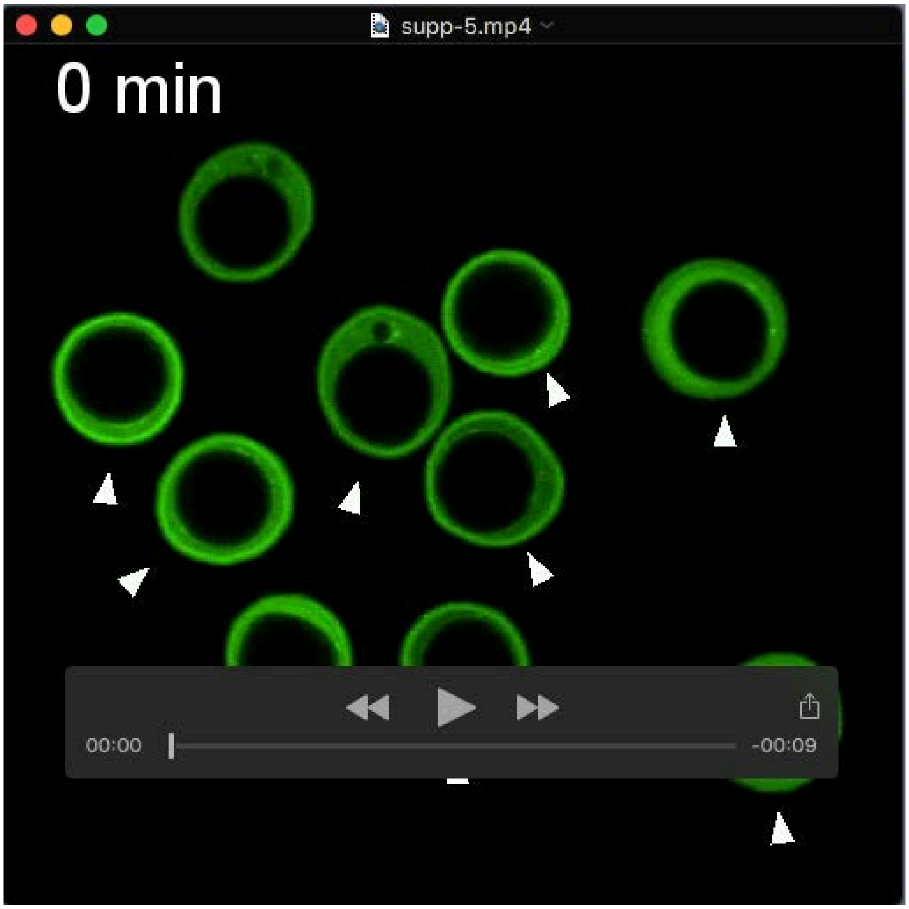

Movie 4. Live imaging of cortical reaction in ovulated oocytes (IVO). Confocal time lapse of IVO oocytes incubated with $SrCl_2$ and LCA-FITC. Arrowheads indicate responding cells.

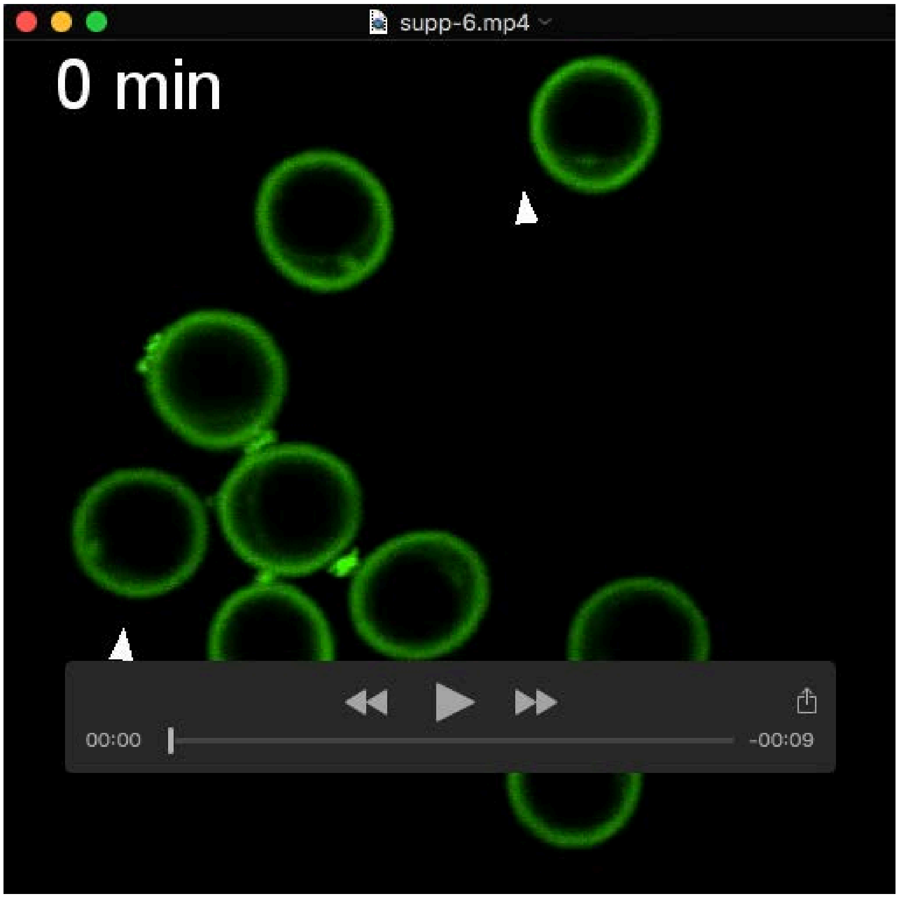

Movie 5. Live imaging of cortical reaction in CZB *in vitro* matured oocytes (CZB IVM). Confocal time lapse of CZB IVM oocytes incubated with $SrCl_2$ and LCA-FITC. Arrowheads indicate responding cells.

Biology Open • Supplementary information

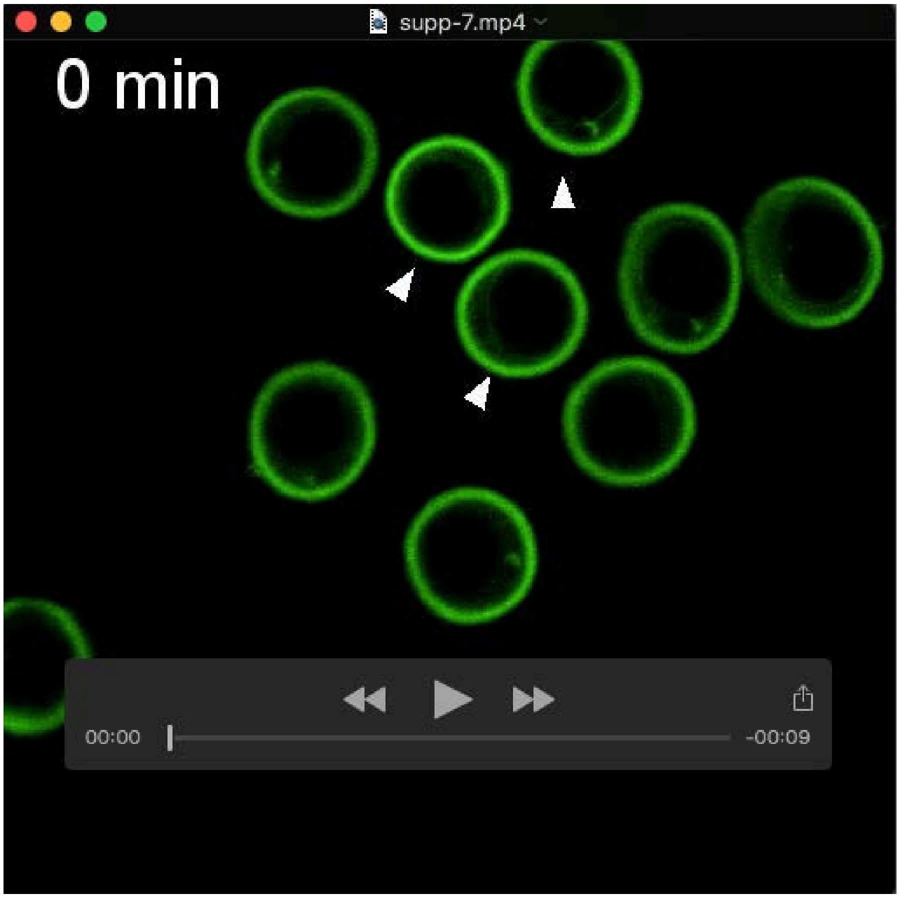

Movie 6. Live imaging of cortical reaction in G-IVF *in vitro* matured oocytes (G-IVF IVM). Confocal time lapse of G-IVF IVM oocytes incubated with $SrCl_2$ and LCA-FITC. Arrowheads indicate responding cells.

Biology Open • Supplementary information
